# Effects of Atmospheric-Pressure N_2_, He, Air, and O_2_ Microplasmas on Mung Bean Seed Germination and Seedling Growth

**DOI:** 10.1038/srep32603

**Published:** 2016-09-01

**Authors:** Renwu Zhou, Rusen Zhou, Xianhui Zhang, Jinxing Zhuang, Size Yang, Kateryna Bazaka, Kostya (Ken) Ostrikov

**Affiliations:** 1 School of Chemistry, Physics and Mechanical Engineering, Queensland University of Technology, Brisbane, Queensland 4000, Australia; 2 Fujian Key Laboratory for Plasma and Magnetic Resonance, School of Physics Science and Technology, Xiamen University, Xiamen 361005, China; 3 Department of Chemical and Biochemical Engineering, College of Chemistry and Chemical Engineering, Xiamen University, Xiamen 361005, China; 4 CSIRO-QUT Joint Sustainable Materials and Devices Laboratory, Commonwealth Scientific and Industrial Research Organisation, P. O. Box 218, Lindfield, NSW 2070, Australia

## Abstract

Atmospheric-pressure N_2_, He, air, and O_2_ microplasma arrays have been used to investigate the effects of plasma treatment on seed germination and seedling growth of mung bean in aqueous solution. Seed germination and growth of mung bean were found to strongly depend on the feed gases used to generate plasma and plasma treatment time. Compared to the treatment with atmospheric-pressure O_2_, N_2_ and He microplasma arrays, treatment with air microplasma arrays was shown to be more efficient in improving both the seed germination rate and seedling growth, the effect attributed to solution acidification and interactions with plasma-generated reactive oxygen and nitrogen species. Acidic environment caused by air discharge in water may promote leathering of seed chaps, thus enhancing the germination rate of mung bean, and stimulating the growth of hypocotyl and radicle. The interactions between plasma-generated reactive species, such as hydrogen peroxide (H_2_O_2_) and nitrogen compounds, and seeds led to a significant acceleration of seed germination and an increase in seedling length of mung bean. Electrolyte leakage rate of mung bean seeds soaked in solution activated using air microplasma was the lowest, while the catalase activity of thus-treated mung bean seeds was the highest compared to other types of microplasma.

Non-equilibrium low temperature plasmas have been attracting significant attention in material fabrication^1^,^2^,^3^,^4^ and more recently in medicine and biotechnology for their ability to induce desirable biochemical responses in living organisms, with potential applications ranging from selective cancer treatment^5^,^6^, wound healing^7^, surface and solution disinfection and decontamination^8^, to sustainable agriculture^9^,^10^,^11^,^12^,^13^. In the case of the latter, the non-ionizing low-level radiation and numerous reactive species, including reactive oxygen and nitrogen species (ROS and RNS) generated by plasma can be used to induce desirable changes in a broad spectrum of developmental and physiological processes in plants, improving seed resistance to stress and diseases, modifying seed coat structures, increasing the permeability of seed coats, and stimulating seed germination and seedling growth^14^,^15^,^16^. These desirable effects were demonstrated in several types of commercially significant food plants for human and animal consumption, such as *wheat*^11^,^17^, *barley*^18^, *tomato*^12^,^19^, *soybean*^10^,^20^ and *thale cress (Arabidopsis thaliana*)^21^. For example, recent studies by Koga *et al*. showed that a single 3-minute treatment of dry seeds of *Arabidopsis thaliana* led to growth acceleration in all the growth stages, including shorter harvest period, a considerable increase in total seed weight, an increase in each seed weight, and a substantial increase in seed number^21^. Although the specific mechanisms by which plasma-generated physical and chemical effects influence the metabolic activity of the seed or the plant remain poorly understood, changes in morphological and sowing features of seeds^11^, dehydrogenase activity, superoxide dismutase (SOD) and peroxidase activity, photosynthetic pigments, photosynthetic efficiency and nitrate reductase activity have been reported^12^.

One of the key reported advantages of plasma seed and plant treatment is that favorable biological responses can be induced in the absence of potentially environmentally-harmful chemicals, which makes plasma-based treatment a more environmentally-sustainable alternative to traditional chemical pathways used to improve seed performance and crop yield. Li and Jiang *et al*. reported that an 80 W cold RF plasma treatment significantly improved seedling growth, including shoot length, shoot dry weight, root length and root dry weight^10^, and Edward *et al*. reported that the yields of lentils, bean and wheat significantly improved as a result of the cold RF plasma treatment induced oxidzation of seed surface and generated nitrogen containing groups^11^. Zhou and Huang *et al*. showed that the effect of plasma treatment on the specific traits and yield of tomato was voltage-dependent and plasma treatment at 6120 V produced best results^22^. However, to date, the majority of the reported plasma treatment centered on the use of low-pressure radio frequency systems^10^,^23^,^24^, which have some obvious limitations in terms of real-life use, specifically with regard to the environmental and economic costs and processing restrictions associated with vacuum processing. This limitation can be addressed by atmospheric-pressure plasmas that are able to produce a wide range of reactive chemical species and physical effects under vacuum-free conditions. Currently, there is a growing number of research groups in USA^25^, Germany^26^, Japan^27^, Australia^28^ and others, that investigate the use of low-temperature (“cold”) atmospheric-pressure plasmas (CAP) on seed metabolism.

Mung bean (*Vigna radiata* (Linn.) Wilczek.) is an important economic crop in South East Asia. Diseases and abiotic stresses, such as drought, heat, water logging and salinity, can lead to a considerable loss in nutritional quality and economic yield of mung bean^29^. These issues are traditionally addressed through genetic engineering and use of growth-inducing chemicals. However, there are two critical issues associated with the use of conventional antibiotics to treat agriculturally-relevant pathogens. The first one is that, just as the case with human pathogens, excessive antibiotic treatment can induce the development of antibiotic resistance, decreasing the effectiveness of not only this therapy but other therapies that share the same microbial target. The resistant pathogen can also transfer the relevant genes to other pathogenic microorganisms, including those that present danger to animals and humans. The second concern involves the unintentional transfer of the sub-inhibitory quantities of antibiotic to the environment, including other plants, animals and humans. In both cases, replacement of the antibiotic with an alternative therapy is beneficial.

Cold atmospheric plasma (CAP) is a new, promising antibacterial treatment to combat antibiotic-resistant bacteria with synergies that arise from chemical species and physical effects. Matthes *et al*. reported that repeated applications of cold atmospheric pressure plasma on *Staphylococcus aureus* embedded in biofilms did not result in the development of resistance or habituation against plasma applied within short time periods^30^. On the other hand, Mai-Prochnow *et al*. reported that a short plasma treatment (3 min) of *Pseudomonas aeruginosa* embedded into biofilms may lead to the emergence of a small number of surviving cells exhibiting enhanced resistance to subsequent plasma exposure^31^.

This study aims to investigate CAP treatment as potential means for enhancement of productivity, specifically seed germination and seedling growth of mung bean crop. Using a custom-built system (Fig. 1), the mechanisms of CAP interactions with the mung bean at different stages of bean development will be studied.

## Results

### Seed germination percentage

Figure 2 shows the typical seed morphology and the germination percentage of mung bean seeds treated with different types of plasma as a function of incubation time. The seed germination percentage was strongly dependent on the incubation time and the feed gas used. As the data from repeated experiments suggest, the germination percentage increased with incubation time, which was translated into a line graph (see Fig. 2(e)). Among tested microplasma arrays, the air and O_2_ microplasma arrays were more efficient in enhancing the seed germination, which could be attributed to the relatively high density of reactive oxygen species generated inside air and O_2_ microplasmas^32^. After incubation for 12 h, seed germination rate of mung bean treated with the air plasma reached 80%, significantly higher than that reached by seeds treated with O_2_ microplasma (15%) and He and N_2_ plasmas (below 10%). After incubation for 24 h, the germination percentage of air plasma treated samples reached approximately 95%, whereas the corresponding value for O_2_ plasma treated seeds ascended to 72%. Seeds treated by He and N_2_ plasma had the lowest germination rate of 30%, almost the same as that for control samples. Finally, after incubation for 48 h, almost all of the treated and control mung bean had germinated.

### Seed germination (germination potential and germination index) and seedling growth (plant length and length index)

Germination potential, germination index are the most significant parameters of biological vigor of the seed^10^. Figure 3 shows that germination potential, germination index, plant length and length index of mung bean seeds were influenced by CAP differently depending on the nature of the gas used to generate the plasma. There were no significant differences in germination potential between samples treated with N_2_, He, Air, O_2_ microplasmas and those in the control group (see Fig. 3(a)), since almost all the seeds germinated after 2 days regardless of being treated or not. Figure 3(b) shows that air plasma treatment significantly increases germination index of mung bean seeds from 60 to 95. The germination index of mung bean seeds treated with O_2_ plasma was slightly lower, at 85. Compared with the control, the air and O_2_ plasma treatments significantly increased the germination index by 58.3% and 41.7%, respectively. On the other hand, there was no significant difference between germination indices of seeds treated with He or N_2_ plasma and that of the control. Overall, air plasma treatment produced the most favorable combination of germination potential, germination rate and germination index of mung bean seeds, suggesting that the cocktail of reactive species produced by this type of plasma under these experimental conditions is best suited to promoting seed germination outcomes of mung bean.

Figure 3(c) shows the effect of CAP treatment on the length of mung bean sprouts as a function of gas used for plasma treatment. Sprouts grown from air plasma-treated seeds had achieved the longest plant length within the incubation time (24–96 h). The plant growth of O_2_ plasma-treated samples was slower, while the N_2_ and He plasma-treated samples displayed sprout lengths similar to those grown from control seeds. After 24 h of incubation time, the plant length of air plasma treated samples reached approximately 10 mm, 3–5 mm longer than that of mung bean treated with other types of plasmas or the control. With the increase of incubation time to 96 h, plants within the air and O_2_ plasma-treated groups reached 67.5 mm and 47.4 mm in length, respectively, while He plasma and N_2_ plasma-treated samples displayed only marginally higher plant lengths than those in control group. Figure 3(d) shows that the respective length indices of mung beans treated by the O_2_, N_2_ and He plasma were 72.3%, 25.1% and 24.9% higher compared to the control, while the corresponding value for air plasma treatment was estimated to be approximately three times that of the control. These results indicate that seeds treated by air discharge not only had better germination performance, but also had a higher growth activity and length index.

### pH value of the plasma treated solution

The pH values of the seed-containing solutions after 10 min of plasma treatment with N_2_, He, air, and O_2_ as feed gas, were measured, as shown in Table 1. All the plasma treatments were performed at V_P_ = 4.5 kV. Treatments with atmospheric-pressure air and N_2_ plasma arrays resulted in a slight decrease in the pH value of the solution. This was attributed to the effects of nitric and nitrate acids produced from the reaction of H_2_O molecules with NO_X_ species, which were generated in the air microplasmas. The pH values of the solutions treated by O_2_ and He microplasma arrays increased only slightly. One possible explanation is that energetic collisions of electrons with water vapor molecules can result in the formation of OH species in water and thus lead to an increase in the pH value^32^,^33^. Previous studies also showed that mung bean seeds treated by slightly acidic electrolyzed functional water presented faster growth than those treated with tap water due to the low electrolyte leakage rate and high catalase activity observed in the former^34^.

### Concentration of plasma-generated H_2_O_2_ molecules and nitrogen-containing species

The potential of N_2_, He, Air and O_2_ plasma treatments to induce changes in the concentration of H_2_O_2_ radicals in distilled water was investigated as a function of the treatment time (Fig. 4(a)). All atmospheric-pressure microplasmas used in this experiment were generated at a V_P_ of 4.5 kV, corresponding to a discharge power of 25 W. Overall, the H_2_O_2_ concentration increased with the duration of plasma treatment time. This increase was attributed to the high electron density, energy of the plasma and long life time of the excited species that facilitate the energy transfer between the excited plasma species and water molecules, leading to H_2_O_2_ formation (*e^−^ + H_2_O → • H + •OH + e^−^, •OH + •OH → H_2_O_2_)^35^. Among these four types of plasmas, the air microplasma treatment showed the highest H_2_O_2_ concentration (17.4 mg/liter) in the 10 min plasma-treated solution, while the H_2_O_2_ concentrations in the O_2_, He and N_2_ microplasma-treated solution were relatively low (7.9 mg/liter, 1.2 mg/liter and 0.5 mg/liter, respectively). The significantly higher H_2_O_2_ concentration in the sample treated with air microplasma may be due to air discharge being more conducive to the formation of OH radicals. On the other hand, as an electronegative gas, O_2_ discharge results in the formation of an excess of oxygen containing species that can adsorb electrons by direct electron attachment (O_2_ + e^−^ → O_2_^−^) or dissociated attachment (O_2_ + e^−^ → O + O^−^), consuming the electrons that would otherwise participate in H_2_O_2_ formation^36^. In He and N_2_ discharges, H_2_O_2_ molecules are produced solely via the collision between energetic electrons and H_2_O molecules^32^, resulting in a significantly lower concentration of H_2_O_2_ in solutions treated with these microplasmas.

The formation of nitrite (NO_2_^−^) and nitrate (NO_3_^−^) in the plasma-treated solution is illustrated in Fig. 4(b), which shows the production of some long-lived and relatively stable chemical species in water as a result of air plasma treatment^37^. NO_2_^−^ and NO_3_^−^ are formed in plasma-treated water through the dissolution of nitrogen oxides formed in the plasma by gas-phase reactions of dissociated N_2_ and O_2_ or H_2_O^38^. Results show that NO_2_^−^ and NO_3_^−^ were formed in water with the constant rate following zero-order rate kinetics indicating a direct effect of the plasma. Among these four types of plasmas, the air microplasma treatment showed the highest NO_2_^−^ and NO_3_^−^ concentration (1.2 mg/liter) in the 10 min plasma-treated solution, followed by that for N_2_ microplasma, O_2_ microplasma, He microplasma listed in decreasing order. Moreover, along with the formation of NO^−^ and NO_3_^−^ in the plasma-treated water, the dissolution of NO_X_ in water produces H^+^ ions following the reaction NO (aq) + NO_2_ (aq) + H_2_O(l) → NO_2_^−^ + NO_3_^−^ + 2H^+^, NO(aq) + NO_2_(aq) + H_2_O(l) → 2NO_2_^−^ + 2H^+^, which is consistent with the measured pH values^35^,^39^.

### Surface physico-chemical properties of mung bean seeds

Scanning electron microscopy (SEM) images of seed coat surface were used to examine the effect of plasma treatment on the morphological characteristics of mung bean seeds. As shown in Fig. 5, surface structure of seeds changed sharply as a result of air plasma treatment. Figure 5(a) indicates that the surface topography of control bean seeds was comprised of irregular rhoptry-shaped features with the size varying from 1.0 to 3.0 μm. The surface structure of N_2_, He, and O_2_ plasma treated seeds did not undergo dramatic changes, and displayed similar topography to that of control samples. By contrast, the air plasma-treated seeds had an eroded surface, with no significant ridges (Fig. 5(b)). These results indicate that acidic environment caused by air discharge in water may have contributed to the chapping of seed coat. After the air plasma processing, the highly compact surface texture of the seed coat may be more fragile and hence easier to crack in acidic plasma-activated water^40^, which would facilitate the more efficient absorption of water and nutrients^41^, and consequently enhance the germination rate and promote the growth of hypocotyl and radicle of the treated mung bean seed. The wettability of seeds can be reflected by the apparent contact angle which results from a complex interplay between chemical composition and roughness of the surfaces^42^,^43^. The apparent water contact angles on surfaces of the He, N_2_ and O_2_ plasma-treated seeds were very similar to that of the control, at 56.4° (Fig. 5), since these plasma treatments did not significantly alter the topography of the seeds. The smallest apparent contact angle was obtained on surfaces of seeds treated with air plasma (Fig. 5(g)), attributed to the plasma-induced changes to the chemical structure and the surface topography of the seed surface. The resulting increased wetting of the air plasma-treated seeds may be partially responsible for the observed increase in the uptake of water^11^,^13^. Notably, the increase in water absorption is often accompanied by an increased ability to absorb nutrients, which promotes the growth of plant seedlings.

### Effects of H_2_O_2_ concentration on seed germination and seedling growth

As shown in Fig. 6(a), H_2_O_2_ played a positive role in accelerating the germination of mung bean. Compared to the control, the six H_2_O_2_ solutions with concentrations ranging from 0.01% to 0.30% all contributed to higher germination rates within fixed incubation time. However, there was an inverse relationship between H_2_O_2_ concentration and the germination rate. During the first 12 h of incubation, mung bean treated with 0.01%, 0.03% and 0.05% of H_2_O_2_ displayed dramatic increases in germination rate, from fairly low levels to more than 60%, while no germination was observed in the control. After 48 h of incubation, both control samples and H_2_O_2_-treated mung bean germinated entirely. The curves presented in Fig. 6(c) show the relationship between H_2_O_2_ concentration and plant length. When the concentration of H_2_O_2_ was below 0.07%, the treatment was highly conductive to plant growth, and the 0.01% H_2_O_2_ treatment outperformed the others at any incubation time. However, when the concentration of H_2_O_2_ was drastically increased to 0.10% and 0.30%, the growth of mung bean was hindered. As mentioned above, 0.01% H_2_O_2_ solution acted as a significant motivator for both germination rate and plant length of mung bean. To further explore the phenomenon, Fig. 6(b,d) were presented to make comparisons between 0.01% H_2_O_2_ treated and air plasma treated samples with respect to their ability to improve the germination rate and plant length, respectively. While the results of the two treatments were similar, air plasma treatment was slightly more effective, especially in boosting plant growth. The disparity implied that although H_2_O_2_ was a major factor in promoting mung bean germination and growth, other plasma-generated factors may have contributed, with potential yet to be fully explored synergies that may arise from distinct plasma effects. As previously mentioned, in addition to a rich mixture of chemical species, plasma generates photons, electric fields, shock waves, etc^44^. For example, formation of solvated electrons at a plasma-solution interface opens questions about their behaviour in the presence of strong electric fields, as suggested by the blue-shifted absorption spectrum^45^.

### Effects of LNF solutions and air plasma treatment time on seed germination and seedling growth

One well-known fact is that nitrogen-containing species such as NO_3_^−^ and NO_2_^−^ are generated in air plasma^46^. In view of this, it might prove instructive to analyze the effects of nitrogen on plant growth as nitrogen is one of the essential nutrient elements in the plant growth^47^. In this experiment, an aqueous solution containing 0.1–3.0 g/L of NaNO_3_ and NaNO_2_ was used to represent liquid nitrogen fertilizer (LNF) to study the effects of nitrogen on the germination and growth of mung bean, and the results were shown in Fig. 7. Clearly, LNF (0.1 g/L to 3.0 g/L) increased the germination rate of mung bean, with the most significant improvement observed in seed groups treated with solutions containing 0.3 g/L LNF. The influence of LNF on plant length (presented in Fig. 7(b)) was similar to that of H_2_O_2_ (shown in Fig. 6(c)), with solution containing lower concentrations of the fertilized (0.3 g/L LNF) creating the most favorable conditions for mung bean growth. Another point that should be noted is that highly concentrated LNF would be detrimental or even fatal to plant growth. This leads us to the conclusion that nitrogen may play a critical role in the air plasma-stimulated germination and growth of mung bean^46^. The effect of air plasma treatment time on germination rate and plant length was also investigated. Figure 7(c,d) shows the effect of air plasma treatment duration on the germination rates and plant growth of mung bean seeds measured as a function of incubation time. Clearly, both the germination percentage and plant growth were strongly dependent on the air plasma treatment time. Although moderately extending the treatment time led to a significant increase in the germination rate and seedling growth of mung bean, this upward trend was restrained when the air plasma treatment was over 15 min. This is mainly because prolonged plasma treatment might result in an increase in the temperature of the solution, adversely affecting plant growth^48^.

## Discussion

Reports have shown that some reactive species generated in plasma gas phase cannot penetrate the gas-liquid interface (several μm to hundreds of μm) or diffuse into the solution within their short life time during the plasma treatment. In general, only a small portion of species, such as O_3_, H_2_O_2_, H, OH, NO_x_ and HNO_x_, can pass through the gas-liquid interface and enter the solution. Compared with other radicals, H_2_O_2_, NO_x_ and HNO_x_ exist in the solution for a longer period of time^33^,^37^. These reactive oxygen species (ROS) and reactive nitrogen species^47^, as evidenced by recent studies, play an important role in cell proliferation, differentiation and apoptosis and can function as signaling molecules^6^. It was detected in our experiment that air plasma generated RNS radicals (nitrogen oxide (NO_X_) molecules, HNO_X_) was in part responsible for the observed acidification of the solution (pH < 7). Acidification of plasma-activated water contributed to the chapping of the waxy layer in the seed coat (see Fig. 5), which in turn promoted the ability of the treated seeds to absorb water and nutrients, increased the germination of mung bean, and accelerated the growth of hypocotyl and radicle. In addition, mung bean seeds treated by air plasma in water had a lower rate of electrolyte leakage, making it possible for the seeds to maintain relatively high activity^46^. The higher root activity of mung bean sprouts further contributed to the growth of the sprouts. Besides, the acidic solution significantly reduced the number of microorganisms on the coat surface of the seeds, in effect decontaminating the seed, which is beneficial to seed germination^15^,^34^,^49^. The ability of plasma-generated chemical species, such as ROS and RNS, and photons to eradicate pathogenic fungi and bacteria in planktonic and biofilm states is well described in literature^32^.

Nitrogen, in particular, is indispensable for plant growth. Under natural conditions, nitrogen bound in soil mainly exists in four types of compounds - ammonium salts (NH_4_^+^), nitrates (NO_3_^−^), proteins and products of protein decomposition (amino acids, amines, peptides and humus compounds). It is justifiable to assume that a favorable environment for mung bean seed germination might occur in solutions containing a proper source of nitrogen, thus improving the nutritional values of the solutions^50^,^51^. Using LNF as feed can increase nitrogen accumulation in the mung bean plant and improve the activity of nitrate reductase and glutamine synthetase related to nitrogen metabolism and photosynthesis, which contributes to the growth of seedling^52^. However, excessive use of LNF will give rise to the disorder of nitrogen metabolism in bean plants, inhibiting mung bean nodule formation and symbiotic nitrogen fixation^53^. Another reason for the reduced rate of canopy photosynthesis under high nitrogen may be that the overdose of nitrogen produces toxic organic nitride, the presence of which damages plant growth^54^.

The effects of different gas discharge treatment on the electrolyte leakage rate of mung bean were investigated and the results are shown in Fig. 8(a). Among all treated seeds, air plasma-treated samples had the lowest electrolyte leakage rate and therefore the highest metabolic activity, so unsurprisingly their hypocotyls were the longest. Compared with air plasma-treated mung bean seeds, those subjected to O_2_ plasma treatment presented a slightly higher electrolyte leakage rate, while the other two treatments showed little difference to the control in this respect. Figure 8(b) shows the effects of different gas discharge treatment on the catalase activity of mung bean. Catalase can remove H_2_O_2_, and is part of the defense system, so the catalase activity is highly interrelated with the ability of plants to tolerate stress. Low catalase activity would lead to the accumulation of H_2_O_2_ in plant cells as well as disruption of metabolic activity^34^. It is clearly seen from the chart that catalase activity of the air plasma treated seeds was 21.9% higher than the corresponding value for the control, which means that air plasma treatment can benefit plant growth by increasing its ability to resist/tolerate stress.

Generally, not all active oxygen species are detrimental, and not all antioxidants are beneficial. Balancing the production and clearance of reactive oxygen species is vital to the plant’s growth and metabolism and its ability to respond to environmental stresses. After a long history of evolution, plants have formed effective mechanisms of active oxygen scavenging which can be divided into two categories: enzymatic and non-enzymatic. The first group includes such enzymes as superoxide dismutase, peroxidase, glutathione peroxidase and ascorbate peroxidase, whereas the non-enzymatic group includes ascorbic acid, carotenoids and flavonoids^55^. In addition to clearing up the ROS through chemical reactions, these substances can also act as a substrate for the enzyme, boosting the active oxygen scavenging. Since H_2_O_2_ treatment enhances the activity of peroxidase, ascorbate peroxidase and ascorbate oxidase, while reducing abscisic acid and zeatin^56^, plasma treatment that delivers sufficient quantities of exogenous H_2_O_2_ to mung bean seeds may effectively increase the oxygen scavenging ability of the plant and thus increase seed germination rate and promote the growth of mung bean seedlings, as shown in Fig. 6.

Plasma treatment can indeed provide a chemical-free means of stimulating seed germination and plant growth. However, to achieve considerable improvement in agricultural efficiency, the enhancement should be preferably maintained throughout the growth cycle in its entirety, leading to higher productivity, i.e. faster harvest, higher weight per fruit or seed, and more numerous fruit or seeds, as well as higher quality, more nutritious and tasty fruit or seed. Recent evidence suggest that valuable plasma effects are indeed retained throughout the growth cycle and even potentially passed on to future generations via pathways other than genetic mutations^21^.

## Conclusion

In this study, investigations of the seed germination and seedling growth rates of mung bean were performed by using atmospheric-pressure N_2_, He, air and O_2_ microplasma arrays in water. Compared to the O_2_, N_2_ and He microplasma treatment, the air microplasma treatment was more effective in enhancing seed germination and seedling growth of mung bean in aqueous solution. Some exogenous experiments including treatment by H_2_O_2_ solution and LNF solution were performed to study the mechanisms of plasma-generated species interactions with the mung bean. Analysis showed that the ROS and RNS species generated by air plasma in solution played a critical role in the germination and growing process. Our research shows the feasibility and advantages of cold plasma application to seed treatment, and also provides theoretical basis for the utilization and popularization of this technique.

## Methods

**Figure 1:**
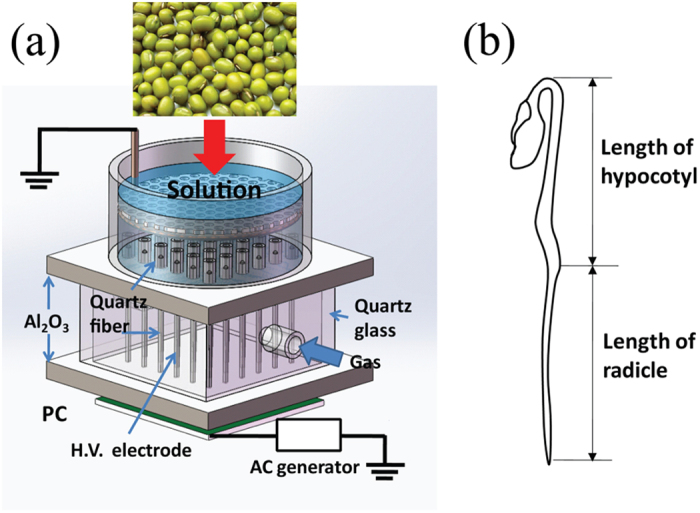
(**a**) The schematic diagram of the experimental setup used in this study. (**b**) The schematic of measuring the morphological indices of mung bean sprout.

**Figure 2:**
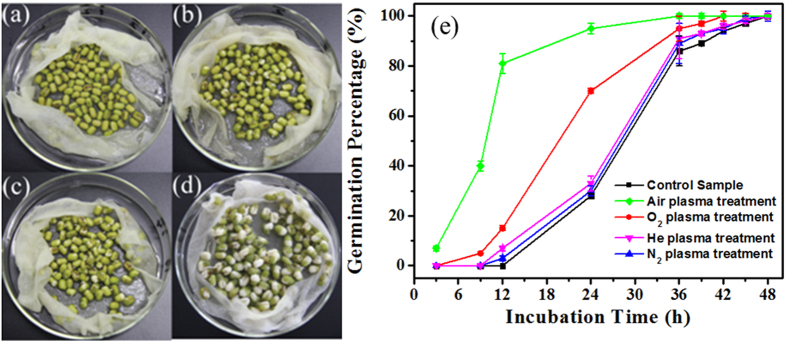
Photographs of air plasma-treated mung bean seeds at different incubation time. (**a**) 0 h, (**b**) 9 h, (**c**) 12 h, (**d**) 24 h, and (**e**) the germination percentage of mung bean seeds treated with He, N_2_, air, or O_2_ plasma as a function of incubation time.

**Figure 3:**
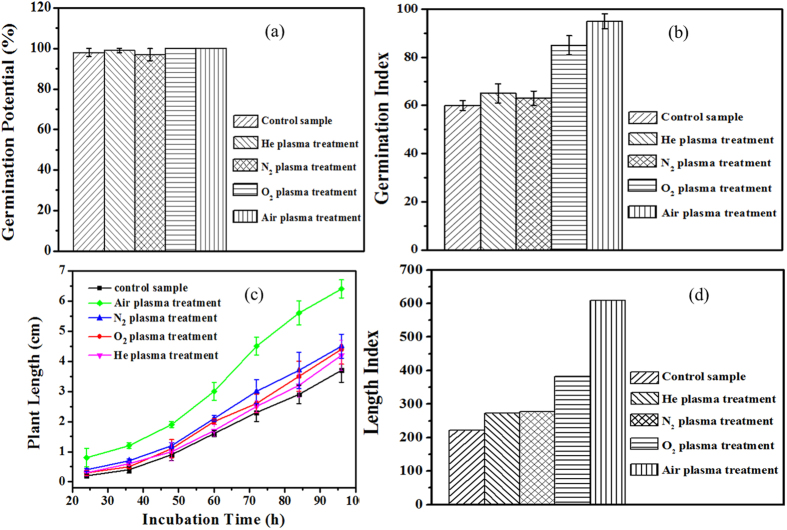
Germination potential (**a**), Germination index (**b**), plant length (**c**) and length index (**d**) of mung bean seeds treated by N_2_, He, Air, O_2_ microplasmas.

**Figure 4:**
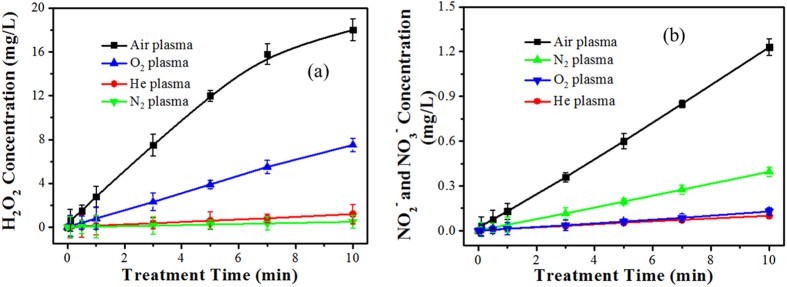
Concentrations of (**a**) H_2_O_2_ and (**b**) NO_2_^−^ and NO_3_^−^ in aqueous solution treated with N_2_, He, Air, or O_2_ microplasmas as a function of the treatment time.

**Figure 5:**
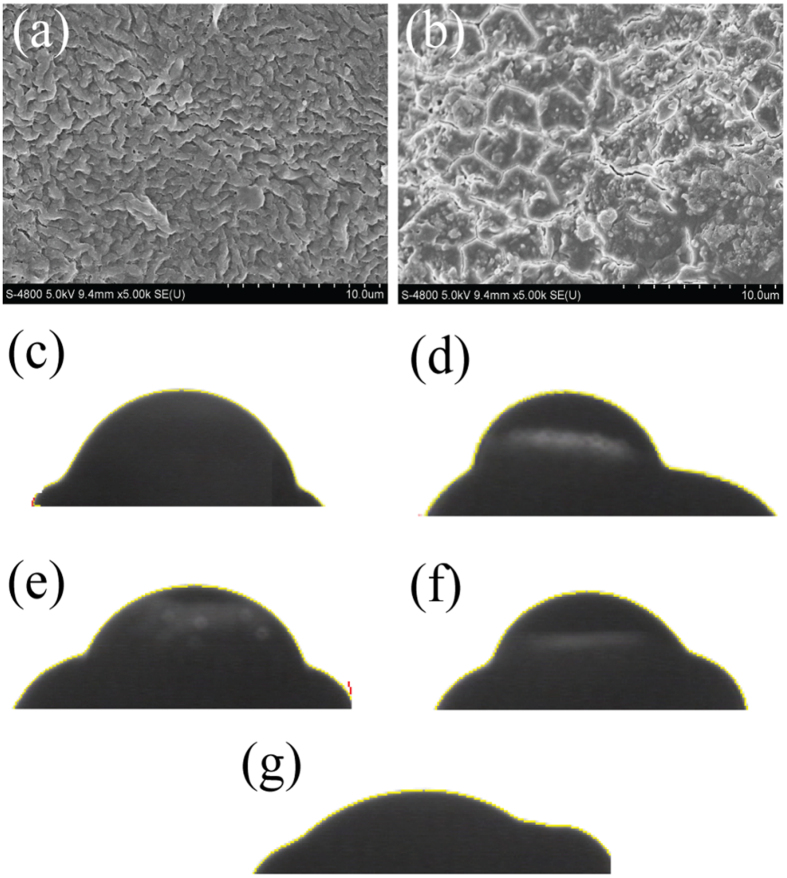
SEM images of the surfaces of (**a**) control mung bean seeds and (**b**) mung bean seeds treated with air plasma. Scale bar is 10 μm. Water droplet deposited on the control (**c**) He plasma-treated (**d**) O_2_ plasma-treated (**e**) N_2_ plasma-treated (**f**) and air plasma-treated (**g**) mung bean seeds.

**Figure 6:**
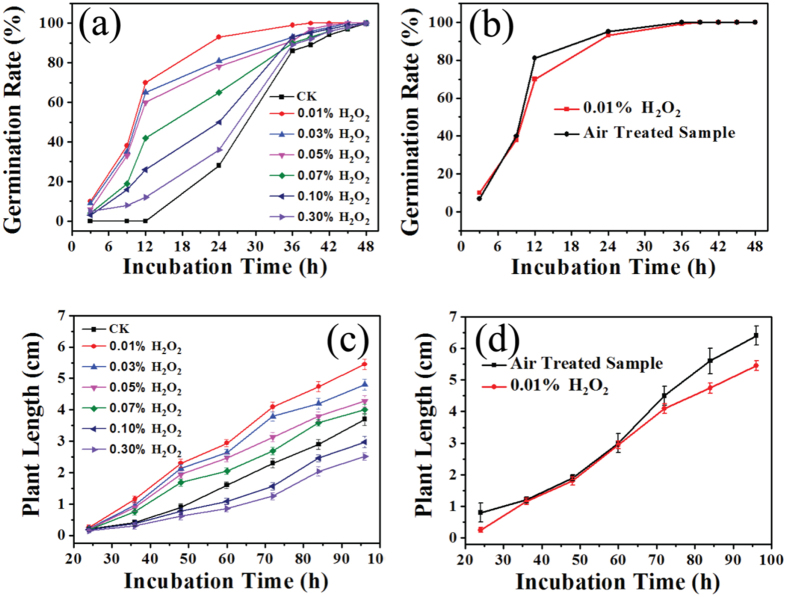
Effect of incubation time on germination rate (**a**) And plant length (**c**) of mung bean treated by H_2_O_2_ solution at different concentration and compared with air treated sample (**b**,**d**) separately.

**Figure 7:**
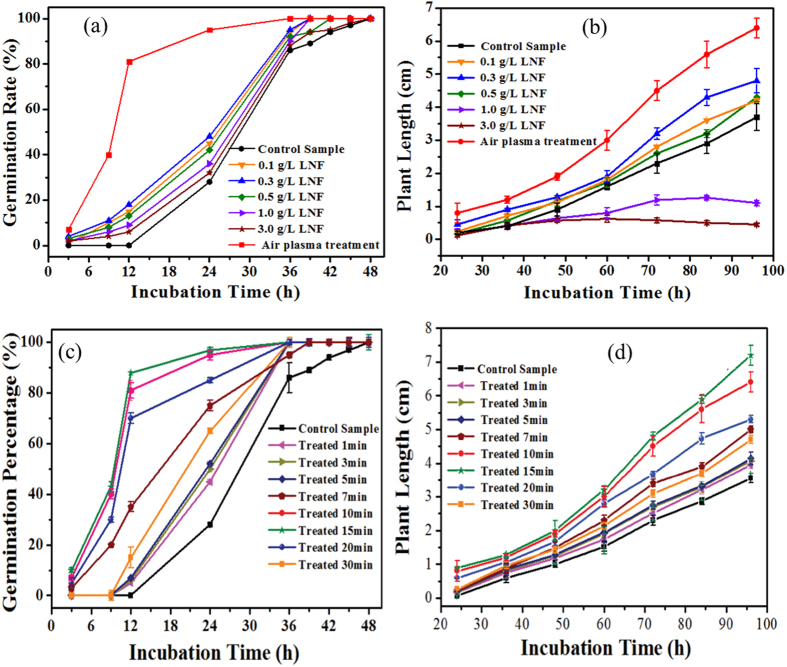
Effect of incubation time on germination rate (**a**) and plant length (**b**) of mung bean treated by liquid nitrogen fertilizers (LNF) of different concentrations; Effect of air plasma treatment duration on germination rate (**c**) and plant length (**d**) of mung bean as a function of incubation time.

**Figure 8:**
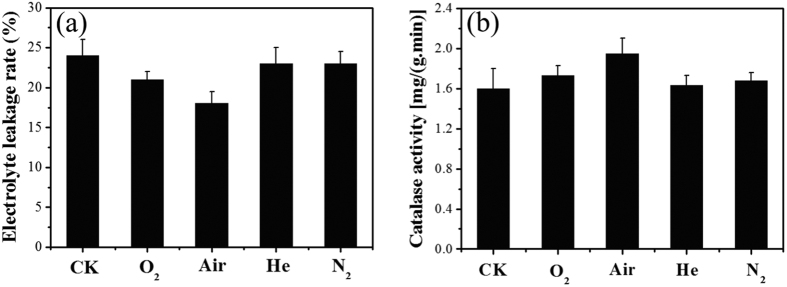
Effects of different gas discharge treatment on the electrolyte leakage rate (**a**) and catalase activity (**b**) of mung bean.

Atmospheric-pressure microplasma array^32^,^57^ is used to treat mung bean seeds in aqueous media, as shown in Fig. 1(a). The feed gases, including He, N_2_, artificial air (‘air’), and O_2_ are added into the 36 microplasma jet units at the flow rate of 2.0 standard liter per minute (SLM). The aqueous solution containing mung bean seeds acts as the grounded electrode. The power supply generates bipolar AC output with the peak voltage (V_P_) of 0–20 kV at an AC frequency of 9.0 kHz. The discharge power can be calculated by a Lissajous figure formed with the charges across the capacitor and the applied voltage across the discharge chamber. In this study, all plasma treatments of mung bean seeds in solution are performed by using the atmospheric microplasma arrays at V_P_ = 4.5 kV, corresponding to the discharge power of 25 W.

Seed treatments were carried out at the Institute of Physics and Mechanical & Electrical Engineering, Xiamen University, Xiamen, China (118°06′E, 24°27′N), from March to September, 2015. 100 uniform seeds of mung bean (obtained in Nanjing City, Jiangsu, China) were overspread on a filter screen which was placed 1 cm above the microplasma jet units in the plasma processing system. The seeds were then exposed to inductive air plasma generated in solution with Dielectric Barrier Discharge (DBD) for 10 min. Meanwhile, the same number of seeds in the control group were also subjected to the same plasma reactor and feed gas flux for 10 min in the absence of plasma. After 10 min of plasma treatment, the treated seeds were placed on the filter cloth in 9 cm petri dishes and 10 mL of distilled water was added into each dish to create germinating conditions. After that, these samples were incubated in a light incubator at the temperature of 25 °C. During the germination and growth, 5 mL of distilled water was added daily to each petri dish to keep sufficient moisture for germination. The germination percentage was recorded every 3 hours for 4 days. The morphological measurements of mung bean sprouts were performed at the 12 h intervals after germination began. The total length of mung bean sprouts, including the length of hypocotyls and the length of radicles, was measured by a ruler, as shown in Fig. 1(b). Every reported measurement represents the average length of 25 sprouts per treatment group.

The concentration of hydrogen peroxide in the plasma-treated water was determined by color forming reactions and spectrophotometric measurements. When titanium oxysulfate (TiOSO_4_) reacts with H_2_O_2_, a yellow-colored complex (pertitanic acid) was formed and UV–Vis measurement was done at 407 nm to colorimetrically determine the concentration of H_2_O_2_ (TiO^2+^ + H_2_O_2_ → [TiO(H_2_O_2_)]^2+^)^38^,^58^. For nitrite and nitrate detection, the well-known Griess assay was used to estimate the concentrations of nitrates (nitrites are first reduced to nitrates), which can react with Griess Reagents to form a deep purple azo compound whose absorption at 550 nm can be measured^58^,^59^. Electrolyte leakage rate^60^ and catalase activity^61^ in seeds were recorded immediately after air plasma treatment. It should be noted that all the seed experiments reported in this letter were planned as a completely randomized design with three replications, and the results are consistent under the same experimental conditions. Several parameters were used to describe the statistical characteristics of seeds:


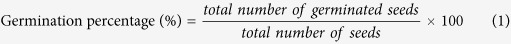



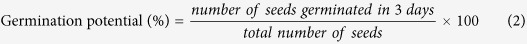



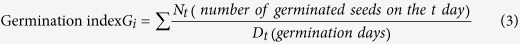



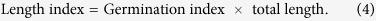


## Additional Information

**How to cite this article**: Zhou, R. *et al*. Effects of Atmospheric-Pressure N_2_, He, Air, and O_2_ Microplasmas on Mung Bean Seed Germination and Seedling Growth. *Sci. Rep.* **6**, 32603; doi: 10.1038/srep32603 (2016).

## Figures and Tables

**Table 1:** The pH values of the solutions after 10 min of plasma treatment with N_2_, He, air, and O_2_ as feed gas.

Discharge gas	Air	O_2_	N_2_	He
pH value of solution	5.1	7.2	6.8	7.5
